# Supplementary material for: Genomic Insights into Cyanide Biodegradation in the Pseudomonas Genus
Source: Int J Mol Sci. 2024 Apr 18;25(8):4456. doi: 10.3390/ijms25084456 (PMC11049912; doi:10.3390/ijms25084456)
Supplement: Supplementary file 1 [file ijms-25-04456-s001.zip › Table S8.pdf]

**Table S5.** Results of the pan-genomic analyses performed in this work.

| | *P. oleovorans* | *P. fluorescens* | *P. monteilii* | *Pseudomonas* |
|---|---|---|---|---|
| Core | 2736 (30.4%) | 1091 (3.7%) | 3508 (30.9%) | 333 (0.3%) |
| Dispensable clusters | 4191 (46.5%) | 11727 (39.5%) | 4564 (40.2%) | 40382 (37.7%) |
| Strain-specific clusters | 2085 (23.1%) | 16876 (56.8%) | 3288 (28.9%) | 66404 (62%) |
| Total clusters | 9012 | 29694 | 11360 | 107119 |
| Number of genomes | 18 | 31 | 17 | 143 |
