# Supplementary figures and images for: Genomic Insights into Cyanide Biodegradation in the Pseudomonas Genus
Source: Int J Mol Sci. 2024 Apr 18;25(8):4456. doi: 10.3390/ijms25084456 (PMC11049912; doi:10.3390/ijms25084456)

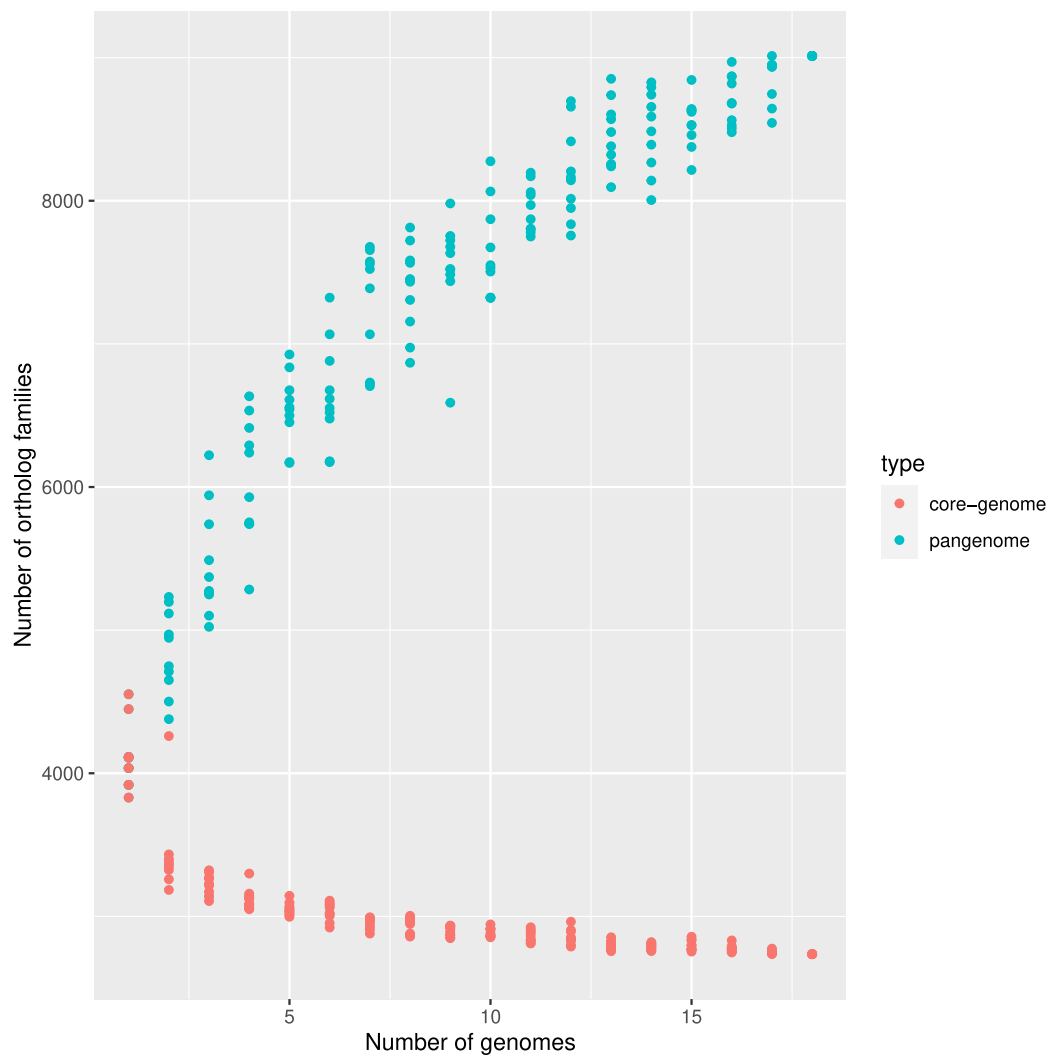

Supplement: Supplementary file 1 [file ijms-25-04456-s001.zip › Figure S1.pdf]

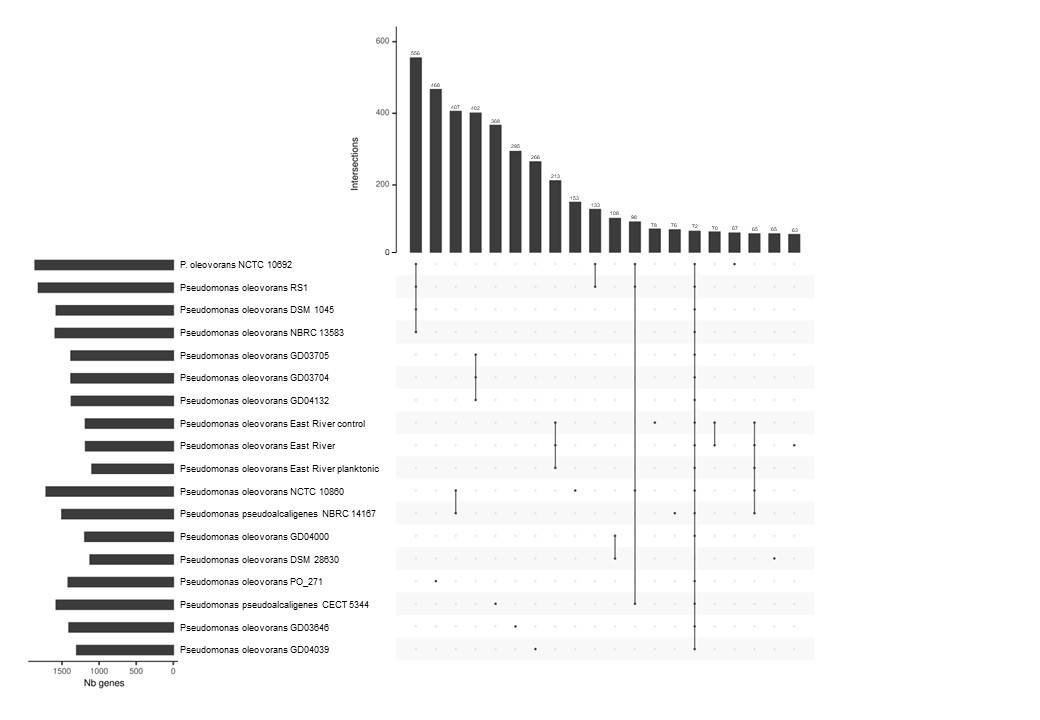

Supplement: Supplementary file 1 [file ijms-25-04456-s001.zip › Figure S2.jpg]

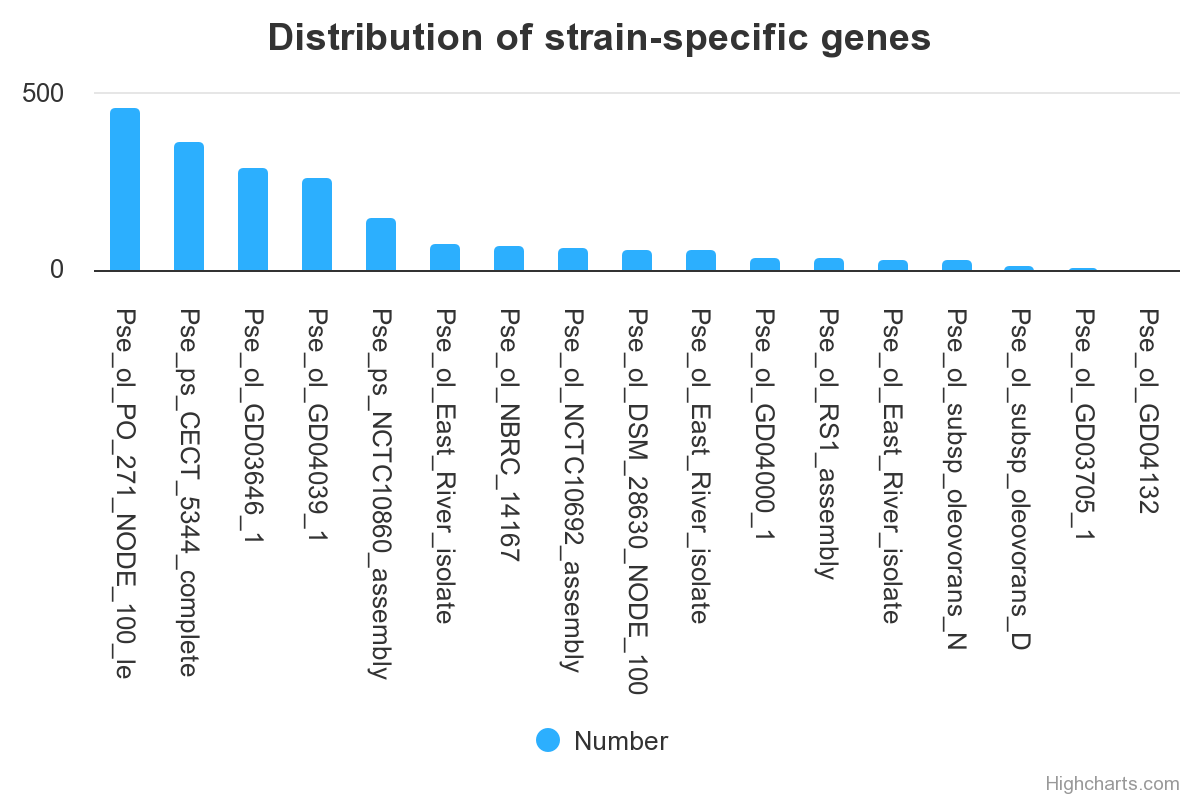

Supplement: Supplementary file 1 [file ijms-25-04456-s001.zip › Figure S3.png]

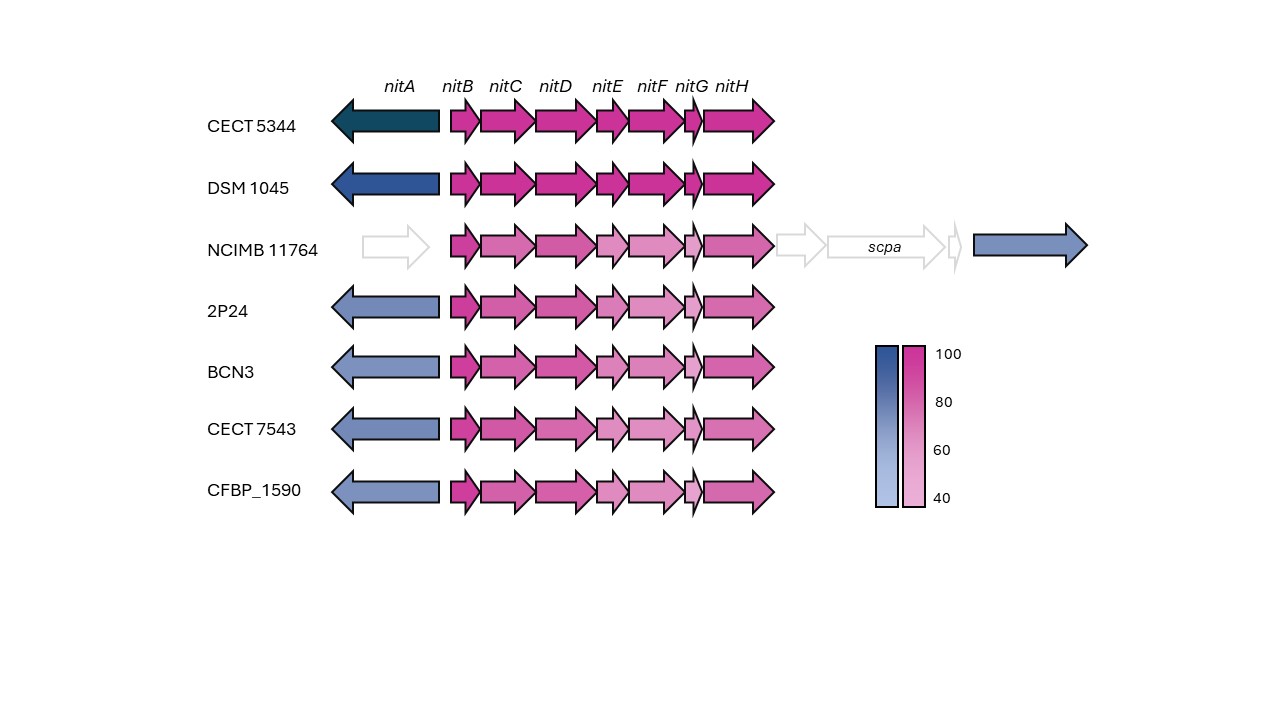

Supplement: Supplementary file 1 [file ijms-25-04456-s001.zip › Figure S6.jpg]

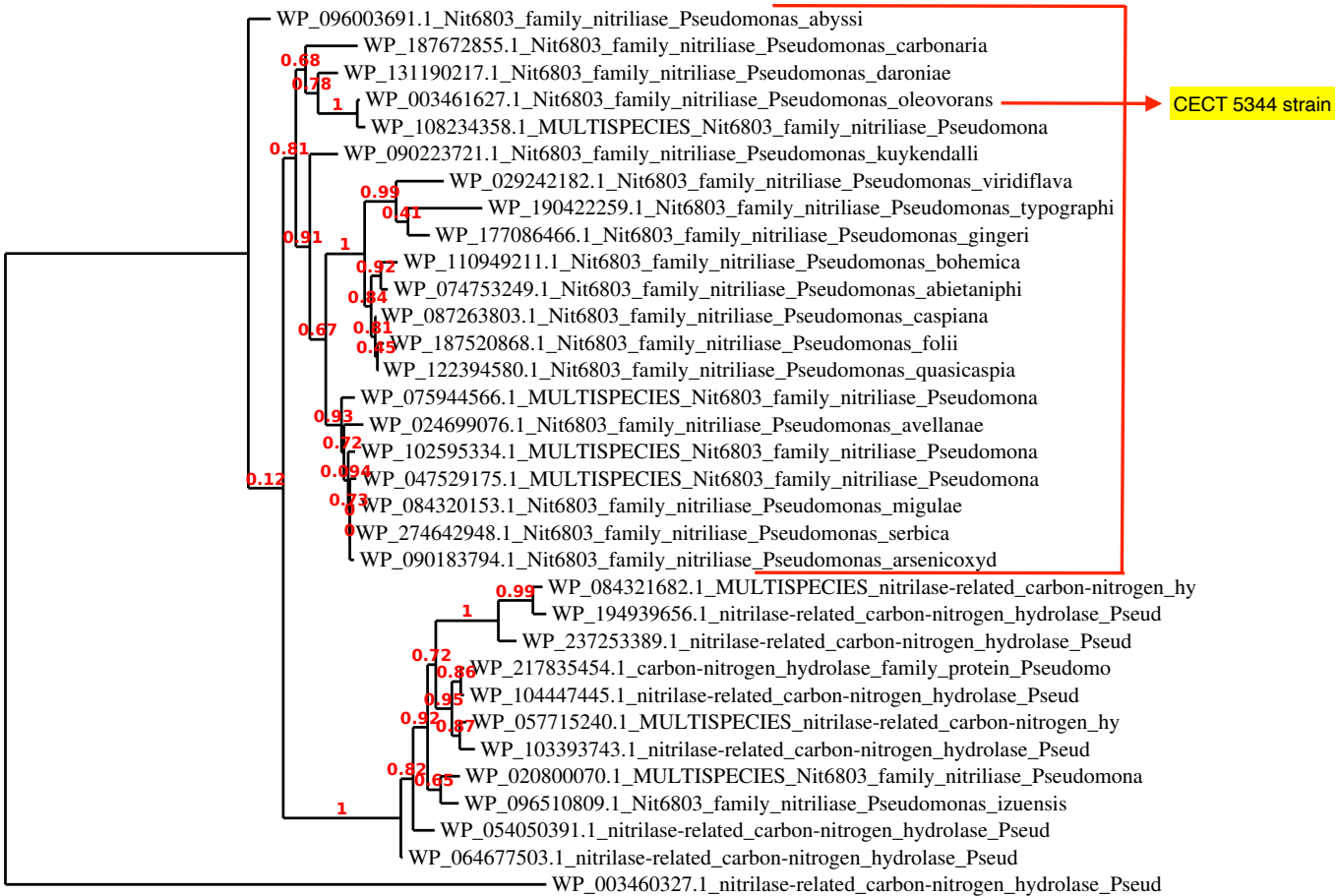

Supplement: Supplementary file 1 [file ijms-25-04456-s001.zip › Figure S7.pdf]
